# An exploration of the attitudes and beliefs of rural women towards skilled birth attendants in Nepal

**DOI:** 10.1186/1753-6561-6-S4-P53

**Published:** 2012-07-09

**Authors:** S Forrest

**Affiliations:** 1 University of Dundee, UK

## Introduction

An exploration of the attitudes and beliefs of rural women towards skilled birth attendants in Nepal. This research project aims to identify factors that contribute to rural women’s decision to use SBAs during childbirth, the factors surrounding that decision and the barriers identified so future policy makers can make informed, critical decisions.

## Methods

Qualitative research was considered most appropriate to understand the attitudes and beliefs of rural women. Semi-structured, one to one interviews were conducted on 12 participants; 4 pregnant women/delivered within 1 year, 4 SBAs and 4 FCHVs/TBAs to gauge a variety of opinions. A purposive approach was used as well as convenience sampling and snowball sampling from the district hospital, various methods of recruitment allowed triangulation and improved validity of results. The same female translator was used throughout to improve reliability of results. Analysis involved using a thematic coding system. Limitations of research resulted from financial and time constraints, small sample size, language barrier and potential for bias.

## Results

The findings were identified from themes emerging from the research and then categorised into subthemes. The Broad general themes identified were:

• Factors encouraging the use of SBAs

• Factors discouraging the use of SBAs

• The benefits/disadvantages of home delivery

• The socio-cultural factors that influence women’s decision

## Conclusions

The results supported some of current research that has been done in literature to suggest the barriers rural women face, however findings of this research report also illustrate the specific challenges government face and the fundamental flaws that need to be addressed. Low levels of female education plays a key role in underutilisation of SBA services through lack of knowledge and awareness, women’s poor social status and power of decision making leading to deprived opportunities. Strategies to improve education, financial/human resource problems and transport were then critically examined.

